# 2,6-Dichloro-7-isopropyl-7*H*-purine

**DOI:** 10.1107/S160053681201879X

**Published:** 2012-05-02

**Authors:** Nikola Hloušková, Michal Rouchal, Marek Nečas, Robert Vícha

**Affiliations:** a Department of Chemistry, Faculty of Technology, Tomas Bata University in Zlin, Nám. T. G. Masaryka 275, Zlín, 762 72, Czech Republic; b Department of Chemistry, Faculty of Science, Masaryk University, Kamenice 5, Brno-Bohunice, 625 00, Czech Republic

## Abstract

In the title mol­ecule, C_8_H_8_Cl_2_N_4_, the essentially planar imidazole and pyrimidine rings [maximum deviations of 0.0030 (15) and 0.0111 (15) Å, respectively] make a dihedral angle of 1.32 (8)°. In the crystal, the fused-ring systems are stacked approximately parallel to the *bc* plane, with a centroid–centroid distance between inversion-related pyrimidine rings of 3.5189 (9) Å.

## Related literature

For the synthesis, see: Oumata *et al.* (2008 ▶). For biological activity of some purine derivatives, see: Legraverend & Grierson (2006 ▶). For the selective synthesis of N7-substituted purines, see: Kotek *et al.* (2010 ▶). For related structures, see: Rouchal *et al.* (2009*a* ▶,*b* ▶, 2010 ▶).
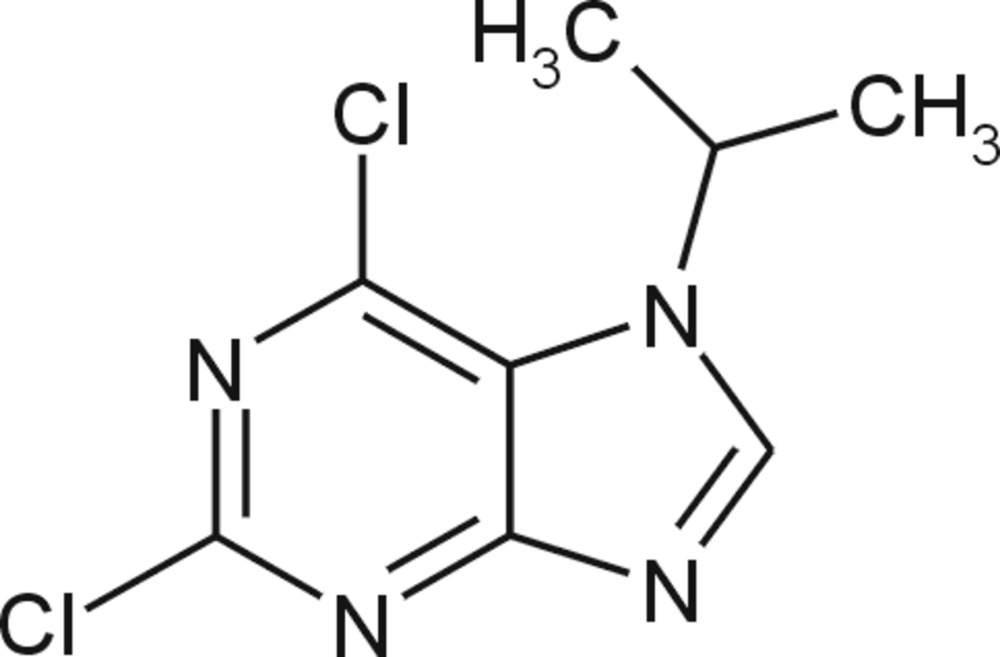


## Experimental

### 

#### Crystal data

C_8_H_8_Cl_2_N_4_
*M* *_r_* = 231.08
Triclinic, 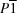

*a* = 7.0146 (5) Å
*b* = 8.2862 (6) Å
*c* = 8.9686 (7) Å
α = 70.499 (7)°
β = 83.820 (6)°
γ = 74.204 (6)°
*V* = 472.75 (7) Å^3^
*Z* = 2
Mo *K*α radiation
μ = 0.65 mm^−1^
*T* = 120 K
0.40 × 0.40 × 0.20 mm

#### Data collection

Oxford Diffraction Xcalibur Sapphire2 diffractometer
Absorption correction: multi-scan (*CrysAlis RED*; Oxford Diffraction, 2009 ▶) *T*_min_ = 0.933, *T*_max_ = 1.000
2825 measured reflections
1656 independent reflections
1419 reflections with *I* > 2σ(*I*)
*R*_int_ = 0.011

#### Refinement

*R*[*F* ^2^ > 2σ(*F* ^2^)] = 0.023
*wR*(*F* ^2^) = 0.061
*S* = 1.05
1656 reflections
129 parameters
H-atom parameters constrained
Δρ_max_ = 0.26 e Å^−3^
Δρ_min_ = −0.24 e Å^−3^

### 

Data collection: *CrysAlis CCD* (Oxford Diffraction, 2009 ▶); cell refinement: *CrysAlis CCD*; data reduction: *CrysAlis RED* (Oxford Diffraction, 2009 ▶); program(s) used to solve structure: *SHELXS97* (Sheldrick, 2008 ▶); program(s) used to refine structure: *SHELXL97* (Sheldrick, 2008 ▶); molecular graphics: *ORTEP-3* (Farrugia, 1997 ▶) and *Mercury* (Macrae *et al.*, 2008 ▶); software used to prepare material for publication: *SHELXL97*.

## Supplementary Material

Crystal structure: contains datablock(s) global, I. DOI: 10.1107/S160053681201879X/lh5458sup1.cif

Structure factors: contains datablock(s) I. DOI: 10.1107/S160053681201879X/lh5458Isup2.hkl

Supplementary material file. DOI: 10.1107/S160053681201879X/lh5458Isup3.cml

Additional supplementary materials:  crystallographic information; 3D view; checkCIF report

## Figures and Tables

**Fig. 1.**
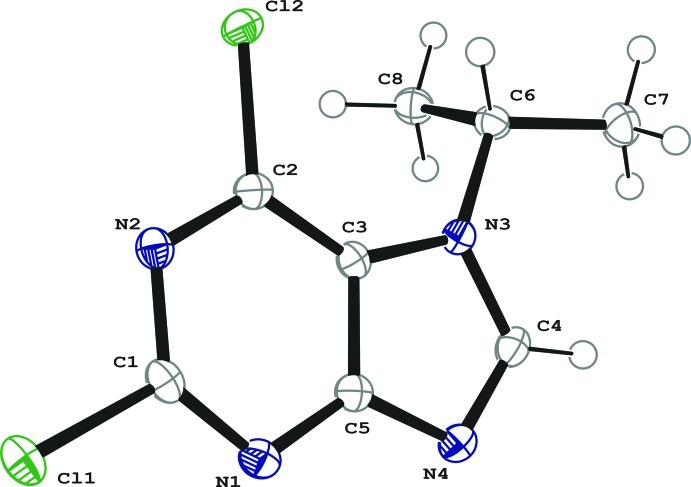
The molecular structure with 50% probability ellipsoids. H-atoms are shown as small spheres at arbitrary radii.

**Fig. 2.**
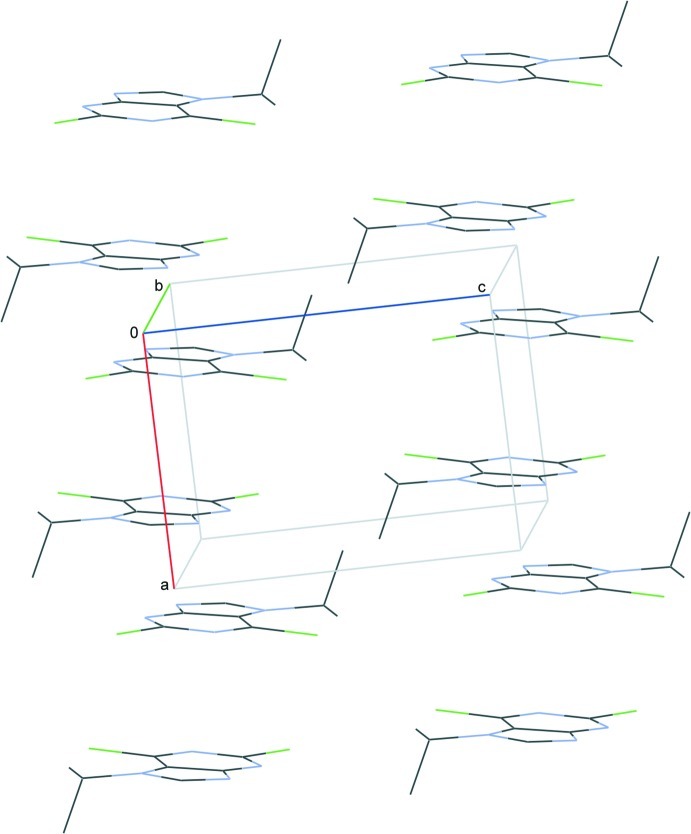
Molecules of title compound stacked along the a-axis. H-atoms have been omitted for clarity.

## supplementary crystallographic information

### Comment

Purines represent a class of compounds with wide range of biological activities. The most of biologically active purines are di-, tri- or tetrasubstituted, usually on the C(2), C(6), C(8) and/or N(9) centers. Moreover, interesting biological properties were also described for some N7-substituted purines (Legraverend & Grierson, 2006). Owing to the relatively small portions of N7 isomer originating within the direct alkylation of purine bases, the selective synthesis of N7-substituted purines was recently described (Kotek *et al.*, 2010). The title molecule was isolated as a side product forming during the synthesis of novel 2,6,9-trisubstituted purine series.

The asymmetric unit of the title compound consists of a single purine molecule (Fig. 1). Both imidazole and pyrimidine rings are essentially planar with maximum deviations from the best plane being 0.0030 (15) Å for C4 (imidazole ring) and 0.0111 (15) Å for C3 (pyrimidine ring). The dihedral angle between the two rings is 1.32 (8)°. In the crystal packing (Fig .2), molecules are stacked parallel to the *bc*-plane. The distance between purine ring atoms (-*x*, 1 - *y*, -*z*) and best plane of adjacent purine ring (*x*, *y*, *z*) varies from -3.4111 (15) Å for N4 to -3.3728 (15) Å for C3. We have already published the structures of some related compounds (Rouchal *et al.*, 2009*a*,*b*; Rouchal *et al.*, 2010).

### Experimental

The title compound was prepared following modified literature procedure (Oumata *et al.*, 2008). To a well stirred solution of 2,6-dichloro-9*H*-purine (4.5 g, 23.8 mmol) in DMSO (50 cm^3^), potassium carbonate (9.9 g, 71.4 mmol) and 2-iodopropane (11.9 cm^3^, 119.0 mmol) were added. The reaction mixture was stirred at 288–291K for 8 h. After that, the mixture was diluted with water (50 cm^3^) and extracted with ether (7 × 15 cm^3^). Collected organic layers were washed with brine (2 × 10 cm^3^), dried over sodium sulfate and evaporated *in vacuo*. Both N7 and N9 isomers were separated from the crude material using column chromatography (silicagel; petroleum ether/ethyl acetate, 1/1, *v*/*v*). 2,6-Dichloro-7-isopropyl-7*H*-purine was obtained as a pale yellow crystalline powder (mp 425–427 K) in minor fraction. The crystal used for data collection was grown by spontaneous evaporation from deuterochloroform at room temperature.

### Refinement

All carbon bound H atoms were placed at calculated positions and were refined as riding with their *U*_iso_ set to either 1.2*U*_eq_ or 1.5*U*_eq_ (methyl) of the respective carrier atoms; in addition, the methyl H atoms were allowed to rotate about the C—C bond.

### Crystal data

**Table:**

C_8_H_8_Cl_2_N_4_	*Z* = 2
*M**_r_* = 231.08	*F*(000) = 236
Triclinic, *P*1	*D*_x_ = 1.623 Mg m^−^^3^
Hall symbol: -P 1	Melting point: 426 K
*a* = 7.0146 (5) Å	Mo *K*α radiation, λ = 0.71073 Å
*b* = 8.2862 (6) Å	Cell parameters from 2034 reflections
*c* = 8.9686 (7) Å	θ = 3.0–27.7°
α = 70.499 (7)°	µ = 0.65 mm^−^^1^
β = 83.820 (6)°	*T* = 120 K
γ = 74.204 (6)°	Block, colourless
*V* = 472.75 (7) Å^3^	0.40 × 0.40 × 0.20 mm

### Data collection

**Table:**

Oxford Diffraction Xcalibur Sapphire2 diffractometer	1656 independent reflections
Radiation source: Enhance (Mo) X-ray Source	1419 reflections with *I* > 2σ(*I*)
Graphite monochromator	*R*_int_ = 0.011
Detector resolution: 8.4353 pixels mm^-1^	θ_max_ = 25.0°, θ_min_ = 3.5°
ω scan	*h* = −8→8
Absorption correction: multi-scan (*CrysAlis RED*; Oxford Diffraction, 2009)	*k* = −9→9
*T*_min_ = 0.933, *T*_max_ = 1.000	*l* = −10→10
2825 measured reflections	

### Refinement

**Table:**

Refinement on *F*^2^	Primary atom site location: structure-invariant direct methods
Least-squares matrix: full	Secondary atom site location: difference Fourier map
*R*[*F*^2^ > 2σ(*F*^2^)] = 0.023	Hydrogen site location: inferred from neighbouring sites
*wR*(*F*^2^) = 0.061	H-atom parameters constrained
*S* = 1.05	*w* = 1/[σ^2^(*F*_o_^2^) + (0.0323*P*)^2^ + 0.0416*P*] where *P* = (*F*_o_^2^ + 2*F*_c_^2^)/3
1656 reflections	(Δ/σ)_max_ < 0.001
129 parameters	Δρ_max_ = 0.26 e Å^−^^3^
0 restraints	Δρ_min_ = −0.24 e Å^−^^3^

### Special details

**Table:**

Geometry. All e.s.d.'s (except the e.s.d. in the dihedral angle between two l.s. planes) are estimated using the full covariance matrix. The cell e.s.d.'s are taken into account individually in the estimation of e.s.d.'s in distances, angles and torsion angles; correlations between e.s.d.'s in cell parameters are only used when they are defined by crystal symmetry. An approximate (isotropic) treatment of cell e.s.d.'s is used for estimating e.s.d.'s involving l.s. planes.
Refinement. Refinement of *F*^2^ against ALL reflections. The weighted *R*-factor *wR* and goodness of fit *S* are based on *F*^2^, conventional *R*-factors *R* are based on *F*, with *F* set to zero for negative *F*^2^. The threshold expression of *F*^2^ > 2σ(*F*^2^) is used only for calculating *R*-factors(gt) *etc*. and is not relevant to the choice of reflections for refinement. *R*-factors based on *F*^2^ are statistically about twice as large as those based on *F*, and *R*- factors based on ALL data will be even larger.

### Fractional atomic coordinates and isotropic or equivalent isotropic displacement parameters (Å^2^)

**Table:**

	*x*	*y*	*z*	*U*_iso_*/*U*_eq_	
Cl1	0.22677 (6)	0.42378 (6)	−0.21906 (5)	0.02126 (13)	
Cl2	0.31193 (6)	0.39565 (5)	0.35468 (5)	0.02020 (13)	
N1	0.2261 (2)	0.71202 (18)	−0.16073 (15)	0.0172 (3)	
N2	0.26395 (19)	0.44137 (17)	0.05831 (15)	0.0160 (3)	
N3	0.27132 (19)	0.83054 (17)	0.17303 (15)	0.0150 (3)	
N4	0.2361 (2)	0.96725 (18)	−0.09060 (15)	0.0175 (3)	
C1	0.2390 (2)	0.5421 (2)	−0.09346 (19)	0.0160 (4)	
C2	0.2758 (2)	0.5246 (2)	0.15927 (18)	0.0153 (4)	
C3	0.2616 (2)	0.7041 (2)	0.10815 (18)	0.0139 (3)	
C4	0.2541 (2)	0.9822 (2)	0.04869 (19)	0.0177 (4)	
H4	0.2549	1.0914	0.0613	0.021*	
C5	0.2397 (2)	0.7924 (2)	−0.05616 (18)	0.0153 (4)	
C6	0.2741 (2)	0.8107 (2)	0.34377 (18)	0.0165 (4)	
H6	0.3683	0.6952	0.3972	0.020*	
C7	0.3474 (3)	0.9569 (2)	0.3651 (2)	0.0245 (4)	
H7A	0.4765	0.9589	0.3115	0.037*	
H7B	0.3608	0.9358	0.4782	0.037*	
H7C	0.2525	1.0708	0.3195	0.037*	
C8	0.0694 (2)	0.8061 (2)	0.41748 (19)	0.0203 (4)	
H8A	−0.0253	0.9186	0.3670	0.030*	
H8B	0.0733	0.7879	0.5310	0.030*	
H8C	0.0282	0.7090	0.4020	0.030*	

### Atomic displacement parameters (Å^2^)

**Table:**

	*U*^11^	*U*^22^	*U*^33^	*U*^12^	*U*^13^	*U*^23^
Cl1	0.0217 (2)	0.0247 (2)	0.0217 (2)	−0.00501 (18)	−0.00236 (17)	−0.01289 (18)
Cl2	0.0284 (3)	0.0158 (2)	0.0155 (2)	−0.00685 (18)	−0.00177 (17)	−0.00235 (17)
N1	0.0152 (7)	0.0196 (8)	0.0164 (7)	−0.0033 (6)	−0.0005 (6)	−0.0062 (6)
N2	0.0136 (7)	0.0170 (8)	0.0182 (7)	−0.0040 (6)	−0.0006 (6)	−0.0064 (6)
N3	0.0158 (7)	0.0140 (7)	0.0155 (7)	−0.0047 (6)	−0.0004 (6)	−0.0041 (6)
N4	0.0188 (8)	0.0152 (8)	0.0177 (7)	−0.0045 (6)	−0.0003 (6)	−0.0038 (6)
C1	0.0109 (8)	0.0211 (9)	0.0188 (8)	−0.0034 (7)	0.0003 (7)	−0.0105 (7)
C2	0.0109 (8)	0.0182 (9)	0.0152 (8)	−0.0035 (7)	0.0004 (6)	−0.0036 (7)
C3	0.0094 (8)	0.0166 (9)	0.0162 (8)	−0.0030 (7)	0.0005 (6)	−0.0062 (7)
C4	0.0161 (9)	0.0137 (8)	0.0220 (9)	−0.0045 (7)	−0.0003 (7)	−0.0038 (7)
C5	0.0103 (8)	0.0184 (9)	0.0162 (8)	−0.0030 (7)	0.0005 (6)	−0.0050 (7)
C6	0.0185 (9)	0.0166 (9)	0.0145 (8)	−0.0036 (7)	−0.0017 (7)	−0.0053 (7)
C7	0.0301 (11)	0.0255 (10)	0.0230 (9)	−0.0119 (8)	−0.0007 (8)	−0.0104 (8)
C8	0.0227 (10)	0.0233 (10)	0.0159 (8)	−0.0066 (8)	0.0016 (7)	−0.0077 (7)

### Geometric parameters (Å, º)

**Table:**

Cl1—C1	1.7437 (16)	C3—C5	1.414 (2)
Cl2—C2	1.7249 (16)	C4—H4	0.9500
N1—C1	1.315 (2)	C6—C7	1.511 (2)
N1—C5	1.343 (2)	C6—C8	1.519 (2)
N2—C2	1.3289 (19)	C6—H6	1.0000
N2—C1	1.339 (2)	C7—H7A	0.9800
N3—C4	1.360 (2)	C7—H7B	0.9800
N3—C3	1.3770 (19)	C7—H7C	0.9800
N3—C6	1.486 (2)	C8—H8A	0.9800
N4—C4	1.318 (2)	C8—H8B	0.9800
N4—C5	1.371 (2)	C8—H8C	0.9800
C2—C3	1.381 (2)		
			
C1—N1—C5	112.46 (14)	N4—C5—C3	110.38 (14)
C2—N2—C1	115.85 (14)	N3—C6—C7	110.35 (13)
C4—N3—C3	105.08 (13)	N3—C6—C8	109.77 (12)
C4—N3—C6	127.26 (14)	C7—C6—C8	112.12 (14)
C3—N3—C6	127.25 (13)	N3—C6—H6	108.2
C4—N4—C5	103.55 (13)	C7—C6—H6	108.2
N1—C1—N2	130.38 (15)	C8—C6—H6	108.2
N1—C1—Cl1	116.32 (12)	C6—C7—H7A	109.5
N2—C1—Cl1	113.29 (12)	C6—C7—H7B	109.5
N2—C2—C3	121.08 (14)	H7A—C7—H7B	109.5
N2—C2—Cl2	116.30 (12)	C6—C7—H7C	109.5
C3—C2—Cl2	122.62 (13)	H7A—C7—H7C	109.5
N3—C3—C2	137.57 (15)	H7B—C7—H7C	109.5
N3—C3—C5	105.65 (13)	C6—C8—H8A	109.5
C2—C3—C5	116.70 (15)	C6—C8—H8B	109.5
N4—C4—N3	115.34 (15)	H8A—C8—H8B	109.5
N4—C4—H4	122.3	C6—C8—H8C	109.5
N3—C4—H4	122.3	H8A—C8—H8C	109.5
N1—C5—N4	126.13 (14)	H8B—C8—H8C	109.5
N1—C5—C3	123.49 (15)		
